# Metabolic priming alters the morphology and metabolism of human dermal fibroblasts

**DOI:** 10.17179/excli2025-8609

**Published:** 2025-10-22

**Authors:** Sónia A. Pinho, Cristina Barosa, Cláudia M. Deus, John G. Jones, Paulo J. Oliveira, Teresa Cunha-Oliveira

**Affiliations:** 1 CNC – UC, Center for Neuroscience and Cell Biology, University of Coimbra, Portugal; 2 CIBB - Center for Innovative Biomedicine and Biotechnology, University of Coimbra, Portugal; 3 PhD Program in Experimental Biology and Biomedicine (PDBEB), Institute for Interdisciplinary Research (IIIUC), University of Coimbra, Portugal; 4 MIA-Portugal, Multidisciplinary Institute of Ageing, University of Coimbra, Portugal

**Keywords:** mitochondria, metabolic priming, bioenergetics, NHDF cells, cell size, ROS-driven adaptation

## Abstract

The metabolic environment provided by the culture medium plays a critical role in shaping cellular function and mitochondrial activity *in vitro*. In this study, we investigated the effects of metabolic priming on the metabolism and morphology of Normal Human Dermal Fibroblasts (NHDFs) by manipulating glucose availability in the culture medium. Our strategy involved transitioning NHDFs from traditional high-glucose medium (HGm) to either a medium with physiological glucose levels (LGm) or a glucose-free, galactose-containing medium (OXm). Prior to cellular characterization, we confirmed the absence of glucose in the culture media and fetal bovine serum using ^1^H nuclear magnetic resonance (NMR) spectroscopy. Given previous observations of elevated reactive species under glucose-free conditions, we explored the cellular adaptations associated with a metabolic shift from glycolysis to oxidative phosphorylation (OXPHOS). Cells cultured in OXm exhibited increased metabolic activity, elevated protein content, and substantial metabolic remodeling. Morphological analysis revealed enlargement of the cell body, cytoplasm, mitochondria, and nuclei, indicative of extensive structural adaptation. Notably, oxygen consumption rate (OCR) nearly doubled within 24 h of exposure to OXm, reflecting a rapid mitochondrial response to metabolic stress. The presence of the antioxidant N-acetyl cysteine (NAC) attenuated this increase, suggesting that redox signaling plays a key role in mitochondrial bioenergetic adaptation. These findings underscore the complex interplay between metabolic context, oxidative stress, and cellular morphology, and highlight the importance of appropriate normalization strategies in metabolic studies.

See also the graphical abstract(Fig. 1).

## Introduction

Cellular metabolism plays a fundamental role in maintaining homeostasis and regulating physiological processes (Li et al., 2023[19]). Disruptions in metabolic pathways are linked to various diseases, including cancer, neurodegenerative disorders, and metabolic syndromes (Liu et al., 2025[22]). *In vitro* models offer a controlled environment in which cellular responses to varying metabolic conditions can be systematically investigated (Hirsch and Schildknecht, 2019[13]; The River Working Group, 2023[47]; Wang et al., 2023[49]). These models not only reduce the reliance on animal studies - addressing ethical and logistical challenges - but also offer human-specific insights when using human-derived cells (Arora et al., 2011[2]; Liu et al., 2018[21]). Even though *in vitro* experiments cannot fully replicate the complexity of living organisms, they are indispensable tools for screening therapeutic compounds, assessing toxicity, and exploring cellular mechanisms (Arora et al., 2011[2]).

Normal human dermal fibroblasts (NHDFs) serve as a valuable *in vitro/ex vivo* model in preclinical research. Derived from human skin tissue, these cells provide an accessible and physiologically relevant system for studying disease mechanisms, particularly those related to mitochondrial function and metabolic adaptation (Madelaire et al., 2022[25]). NHDFs retain key features of human cell physiology, making them well-suited for investigating conditions such as mitochondrial dysfunction and oxidative stress (Juhl et al., 2020[14]). Their ability to reflect *in vivo* metabolic processes enhances their applicability in translational research (Deus et al., 2020[8]; Juhl et al., 2020[14]; Mesdom et al., 2020[28]).

A critical aspect of using NHDFs and other *in vitro* models is ensuring accurate characterization of their metabolic state. Proper characterization is essential for maintaining reproducibility and ensuring that experimental conditions closely mimic physiological interactions. Understanding how fibroblasts adapt to different metabolic environments, including variations in nutrient availability, provides insights into cellular resilience and metabolic plasticity (Batista Leite et al., 2021[4]).

In our previous work (Pinho et al., 2022[38]), we employed a metabolic priming technique by gradually altering glucose availability in the culture medium - from the traditional high-glucose medium (HGm, 25 mM glucose), to a low-glucose medium (LGm), and finally to a glucose-free medium containing 10 mM galactose (OXm). This transition led to mitochondrial remodeling and an increased reliance on oxidative phosphorylation (OXPHOS) for ATP production. Notably, we found that merely reducing glucose availability did not induce the same metabolic adaptations. Lowering glucose to physiological levels altered mitochondrial network dynamics but had no significant impact on overall metabolic activity. These observations underscore the importance of understanding how cells metabolize different sugars and how these pathways influence mitochondrial function.

Unlike glucose, which directly enters glycolysis via hexokinase and is quickly metabolized to produce energy, galactose metabolism is less direct and proceeds through the Leloir pathway, making it less efficient for rapid ATP production. Within the cell, galactose is first phosphorylated by GALK (galactokinase) to form galactose-1-phosphate, which is then converted to glucose-1-phosphate through a series of reactions involving the enzymes GALT (Galactose-1-phosphate uridyltransferase) and GALE (UDP-Galactose-4-Epimerase). Finally, glucose-1-phosphate is transformed into glucose-6-phosphate by phosphoglucomutase (PGM) (Petry and Reichardt, 1998[37]; Coelho et al., 2017[6]). Glucose-6-phosphate then enters the glycolytic pathway, where it is further metabolized to produce pyruvate. However, the rate of galactose metabolism in these initial steps is much slower compared to that of glucose phosphorylation by hexokinase. This limits the rate of pyruvate formation from galactose compared to glucose, making the cells more reliant on OXPHOS for their ATP production to survive and proliferate (Rossignol et al., 2004[43]; Marroquin et al., 2007[27]). As a result, cells grown in a galactose-based medium exhibit metabolic shifts that can provide valuable insights into defective mitochondrial energy production and adaptive responses to stress. Still, there are several aspects of this metabolic priming that are still unexplored.

In this work, we first aimed to confirm the absence of glucose in our culture media and fetal bovine serum (FBS) before proceeding with metabolic characterization. To achieve this, we utilized ^1^H nuclear magnetic resonance (NMR) spectroscopy. We also investigated morphological changes in fibroblasts and explored the cellular mechanisms underlying adaptation to distinct metabolic environments, with a particular focus on the elevated levels of H₂DCF-reactive species previously observed under glucose-free conditions (Pinho et al., 2022[38]).

## Methods

### Cell culture and metabolic priming procedure

NHDF cells (#CC-2511, primary cells, female, 27 years) were purchased from Lonza (Basel, Switzerland) and were used until passage 15, from the initial vial, as recommended by the supplier. NHDF cells were cultured at 37 °C in a humidified atmosphere of 5 % CO_2_ in High-Glucose Medium (HGm; Dulbecco's Modified Eagle's Medium (DMEM D5030, Sigma) supplemented with 4.5 g/L (25 mM) D-glucose, 0.976 g/L (6 mM) L-glutamine, 0.11 g/L (1 mM) sodium pyruvate, 3.7 g/L (44 mM) sodium bicarbonate, 100 U/ml penicillin, 100 μg/ml streptomycin, 250 ng/ml antifungal amphotericin B and 10 % (v/v) FBS, pH 7.2. All cells were passaged by trypsinization at about 90 % confluence.

A subset of cells was gradually adapted to the new media over the course of one passage. On day 1, cells were seeded in 100 mm tissue culture dishes at 37 °C in a humidified atmosphere with 5 % CO₂, using a 75:25 mixture of high-glucose medium (HGm) and the final medium - either OXPHOS-promoting medium (OXm) or low-glucose medium (LGm). These media are compositionally similar to HGm, except for the carbohydrate source: OXm contains 1.8016 g/L galactose (10 mM), and LGm contains 0.9 g/L D-glucose (5 mM), replacing the 25 mM glucose present in HGm.

When cells reached ~50-60 % confluence, the culture medium was replaced with a 50:50 mixture of HGm and final medium (day 2), without trypsinization. Upon reaching ~70-75 % confluence, the medium was replaced again with a 25:75 HGm:final medium mixture (day 3). Finally, at ~90 % confluence, cells were passaged by trypsinization into 100 % final medium (day 4). Before beginning the experimental assays, cells were maintained in 100 % final medium for 48 hours. In accordance with the supplier's recommendation, cells were used for experiments up to a maximum of 15 passages from the initial vial. A step-by-step protocol is available in (Pinho et al., 2025[39]).

### Cell preparation for NMR analysis 

For ^1^H NMR analysis, basal medium DMEM, FBS and standards of pure glucose and galactose prepared with the same concentration, 5 mM, as well as complete culture medium with high and low glucose (HGm and LGm respectively) and glucose-free medium containing galactose, were collected, weighted and stored at -80 ºC. Before analysis, frozen samples were lyophilized. The dried samples were resuspended in 275 μL of 99.9 % deuterated water and a standard of pyrazine solubilized in deuterated water (0.27 mmol/g) was added (25 µL per sample) for metabolite quantification. The samples were loaded into 3 mm NMR tubes.

### Nuclear Magnetic Resonance

^1^H spectra were acquired on a 14.1 T Varian VNMR 600 MHz (Varian Inc., Palo Alto, CA, USA) spectrometer equipped with a 3 mm indirect-detection probe using a standard presaturation pulse sequence (Barosa et al., 2012[3]). Spectra were acquired at 25 ºC, 20 Hz spin rate, with a 45-degree pulse angle, an interpulse delay of 30 s, acquisition time of 3.9 s and 16 free-induction decays (f.i.d.). The spectra were processed with 0.2 Hz line- broadening before Fourier transformation. Processing and integration of ^1^H NMR spectra were performed using NutsPro - NMR Utility Transform Software (Acorn NMR, Inc., Livermore, CA, USA) (Barosa et al., 2012[3]).

### Resazurin reduction

Cell viability was measured through the evaluation of metabolic activity, expressed as the capacity of cellular reduction of resazurin to resorufin by dehydrogenases in viable cells (O'Brien, Wilson et al. 2000[32]). NHDF cells, previously cultured in HGm, LGm and OXm, were seeded at a density of 3,750 cells/well in 96-well fluorescence plates with a final volume of 200 μL. Upon 48 h in culture, a resazurin stock solution (1 mg/mL in PBS 1x) was prepared and the cell culture medium was replaced by resazurin working solution, consisting in 10 µg/mL resazurin in the respective culture medium, HGm, LGm or OXm, in each well (80 μL/well). After 1 h incubation at 37 ºC and 5 % CO_2_ atmosphere, the metabolic activity of cells was measured in a Cytation 3 UV-Vis multi-well plate imaging reader (BioTek, Winooski, VT, USA) using excitation wavelength of 540 nm and emission of 590 nm (Silva et al., 2016[45]). By the end of metabolic activity measurement, cells were prepared for determination of cellular mass through the sulforhodamine B (SRB) assay (next section). Data analyses were performed by GraphPad Prism v.9.0.0 for Windows (GraphPad Software, Boston, Massachusetts USA), RRID:SCR_002798.

### Cellular protein content

Cells that were plated for the Resazurin assay were prepared for the SRB assay, which was conducted to determine cell mass based on cellular protein content (Vichai and Kirtikara, 2006[48]). First, the cell culture medium was removed, and the wells were rinsed with PBS 1x. Next, cells were fixed by adding 1 % acetic acid in 100 % methanol and kept at -20 °C for at least 2 h. Afterwards, the fixation solution was discarded, and the plates were dried at 37 °C. Then, 150 µL of 0.005 % SRB (#S9012-25G, Sigma-Aldrich) in 1 % acetic acid solution was added and incubated at 37 °C for 1 h. The wells were washed with 1 % acetic acid in water and dried. Finally, 100 µL of Tris (pH 10) was added, and the plates were stirred for 15 min before measuring the optical density at 540 nm using a Cytation 3 reader (Biotek Instruments, Winooski, VT, USA). At the end, cells were washed twice with PBS 1x, labelled with bisbenzimide trihydrochloride (Hoechst 33342, # J62134, Alfa Aesar) (0.5 μg/mL in PBS 1x, 20 min at room temperature) and were imaged using an InCell Analyzer automatic fluorescence microscope (GE Healthcare, Chicago, Illinois, USA) equipped with 109/0.45, PlanApo, CFI/60 optics (Nikon, Tokyo, Japan), in DAPI fluorescence detection channel, for 10 fields per well at 10x magnification, covering the whole well. For nuclei segmentation and counting, images were loaded into CellProfiler 4.2.1, RRID:SCR_007358 (Stirling et al., 2021[46]) and analysis was performed as previously reported by us (Pinho et al., 2025[39]).

### Determination of cell area

NHDF cells, previously cultured in HGm, LGm or OXm, were plated at a density of 3,750 cells/well in 96-well fluorescence plates (#353219, Falcon®) with a final volume of 200 μL. After 48 h, cells were incubated for 30 min at 37 ºC with culture medium supplemented with 0.8 µM of CellTracker™ Green CMFDA Dye (CTG) (#C2925, Molecular Probes, Invitrogen, Eugene, OR, USA) with 0.5 µg/mL Hoechst 33342 at (100 µL/well). After probe loading, the culture medium was replaced by new one without dye, sodium bicarbonate or FBS, and cells were imaged in an InCell Analyzer 2200 (GE Healthcare, Chicago, Illinois, USA). Images were acquired in 10 fields per well at 40x magnification (Nikon 10x/0.45, PlanApo, CFI/60) in DAPI and FITC-FITC2 (excitation 475/28 nm; emission 525/48 nm). Analysis of cell, cytoplasm and nuclear area were performed using CellProfiler 4.2.1, RRID:SCR_007358.

The workflow began with the segmentation and counting of nuclei. Images were first pre-processed using illumination correction modules (CorrectIlluminationCalculate and CorrectIlluminationApply) to account for non-uniform background intensity. Nuclei were then segmented using the IdentifyPrimaryObjects module, applied to the illumination-corrected DAPI channel (Stirling et al., 2021[46]; Pinho et al., 2025[39]). 

Following nuclei identification, whole-cell segmentation was carried out using the FITC channel. The pipeline included a second round of illumination correction, followed by the IdentifySecondaryObjects module, which propagated cell boundaries outward from the identified nuclei to delineate full-cell regions.

To define the cytoplasmic area, the IdentifyTertiaryObjects module was used to subtract the nuclear area from the total cell area, thereby isolating the cytoplasmic compartment. 

Quantitative data was exported using the ExportToDatabase module in CellProfiler and opened in SQLite. The resulting tables were saved as .CSV files for downstream analysis. Mean object area measurements were extracted from the parameters Mean_Cells_AreaShape_Area, Mean_Cytoplasm_AreaShape_Area, and Mean_Nuclei_AreaShape_Area, corresponding to whole-cell, cytoplasmic, and nuclear regions, respectively. To normalize area per cell, the mean area values from each independent experiment were divided by the mean number of nuclei identified in the same experiment. As CellProfiler reports area in pixels, values were converted to square micrometers (µm²) using the formula: Area (µm²) = Area (pixels) × (0.4875)², based on a pixel size of 0.4875 µm, as provided in the metadata of the TIFF images acquired with the IN Cell Analyzer 2200.

### Measurement of mitochondrial area

The dye 10-N-nonyl acridine orange (NAO) is used as a mitochondrial probe due to its high affinity for cardiolipin (Rodriguez et al., 2008[42]), a phospholipid that is only found in the inner membrane of mitochondria in mammalian cells. NHDF cells cultured in HGm, LGm or OXm were plated at a density of 3,750 cells/well in 96-well fluorescence plates (#353219, Falcon®) with a final volume of 200 μL. After 48 h, cells were incubated in the dark for 30 min at 37 ºC and 5 % CO_2_ atmosphere with 100 nM NAO (#A1372, Molecular Probes, Invitrogen, Eugene, OR, USA) in each medium without FBS and cells were imaged in an InCell Analyzer 2200 (GE Healthcare, Chicago, Illinois, USA). Images were acquired in 10 fields per well at 40x magnification (Nikon 40x/0.45, PlanApo, CFI/60) in FITC-FITC2 (excitation 475/28 nm; emission 525/48 nm). Analyses of mitochondrial area were performed using CellProfiler 4.2.1, RRID:SCR_007358. To correct for uneven background illumination, the pipeline included the CorrectIlluminationCalculate and CorrectIlluminationApply modules. Fluorescent objects were segmented using the IdentifyPrimaryObjects module, guided by adaptive thresholding defined in the Threshold module. Morphometric and intensity features of the segmented objects were then quantified using the MeasureObjectSizeShape and MeasureObjectIntensity modules, respectively. Additionally, the MeasureImageAreaOccupied module was used to assess the total area occupied by the NAO-stained structures within each image. Data were exported using the ExportToDatabase module in CellProfiler and processed in SQLite. The resulting tables were saved as .CSV files for downstream analysis. Area and intensity measurements were extracted from the parameters Mean_Cells_AreaShape_Area (cell labelled area) and Mean_Cells_Intensity_IntegratedIntensity_CorrNAO (integrated fluorescence intensity of NAO-stained structures). To calculate area per cell, the mean total area from each independent experiment was divided by the corresponding mean cell count. For fluorescence quantification, intensity per cell was determined by dividing the integrated fluorescence intensity by the number of cells, while intensity per area was calculated by dividing the integrated intensity by the total measured area for each experiment.

### Real-time oxygen consumption rate (OCR)

OCR was monitored in real-time using the Resipher system (Lucid Scientific, Atlanta), which operates inside an incubator at 37 °C with a standard 96-well plate. The system consisted of a base station for data storage, a device magnetically attached to an oxygen-sensing lid, and a sterilized lid containing 32 microprobes with oxygen sensors, positioned in columns 3, 4, 9, and 10 of a standard 96-well plate.

**Figure 2:**
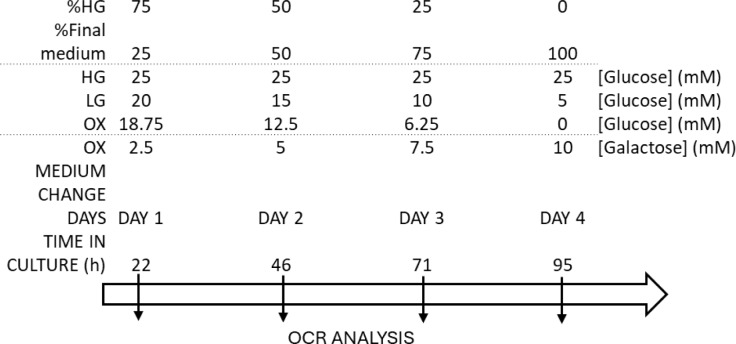
Schematic representation of medium transition from HGm to LGm or OXm using Resipher device. OCR was measured by using a Resipher device (Lucid Scientific). Cells were seeded (2,000 cells/well) in a 96-well plate with 75 % HGm/25 % final medium plus 5 mM NAC (day 1). During 4 days (95 h) medium was changed always in the presence or absence of NAC to 50 % HG/50 % final medium (day 2); to 25 % HG/75 % final medium (day 3) and in day 4 we changed the media to 100 % each final medium (HGm, LGm, OXm).

NHDF cells in HGm were seeded at a density of 660 cells/cm², with 100 µL per well, into the wells in columns 3, 4, 9, and 10 of the 96-well plate. Sterile PBS 1x or sterile MilliQ water was added to the remaining columns to minimize evaporation in the wells containing cells. The adaptation protocol was initiated, as shown in Figure 2(Fig. 2), by transitioning the cells to 75 % HGm/25 % LGm or OXm in a final volume of 150 µL. The plate was gently shaken to homogenize the media. Cells were allowed to adhere for 4-6 h in the CO_2_ incubator. During this period, the Resipher device was acclimated to 37 °C inside the incubator. Once cells had been attached, the Resipher oxygen-sensing lid was placed onto the microplate wells, and the device, along with the microplate, was returned to the incubator to initiate OCR measurements. Cells were incubated for 4 days to allow adaptation to the different media. Every 24 h, the medium was replaced according to the dilution schedule described in Section 1.2.1, using a final volume of 150 µL per well, without passaging the cells to a new plate on day 4. The adaptation process was considered complete once cells had been maintained for at least 24 h in 100 % of their respective final medium.

In parallel, the same procedure was performed using medium supplemented with 5 mM N-acetyl cysteine (NAC). At each 24-hour interval, the culture medium was replaced with the programmed HGm-to-LGm or HGm-to-OXm mixture, supplemented with 5 mM NAC. This allowed metabolic priming to be monitored in the presence or absence of antioxidant treatment.

After the final OCR measurement, cells from both conditions (±NAC) were gently washed with 1× PBS and fixed with 4 % paraformaldehyde (PFA) for 20 minutes at room temperature. Fixed cells were stained with bisbenzimide trihydrochloride (Hoechst 33342; 0.5 µg/mL in 1× PBS; Alfa Aesar, #J62134) for 20 minutes at room temperature and imaged using an InCell Analyzer automatic fluorescence microscope (GE Healthcare, Chicago, IL, USA) equipped with 109/0.45 PlanApo CFI60 optics (Nikon, Tokyo, Japan). Images were acquired in the DAPI channel (excitation: 390/18 nm; emission: 432/48 nm), covering 10 fields per well at 10× magnification to ensure full-well coverage.

For nuclei segmentation and counting, images were loaded into CellProfiler 4.2.1, RRID:SCR_007358, including illumination correction (CorrectIlluminationCalculate + CorrectIlluminationApply) and nuclei segmentation (IdentifyPrimaryObjects) modules. Nuclei were segmented based on the illumination correction image (Stirling et al., 2021[46]; Pinho et al., 2025[39]). The number of nuclei per well was determined by summing the values of Image_Count_Nuclei across all image fields within the same well. This variable is located in the ExpNo_Per_Image table of the database. To normalize the OCR per cell, values were normalized by dividing by the total nuclei count per well.

## Results

Before proceeding with cellular characterization under the different metabolic conditions, we first verified that the powdered medium and FBS were free of glucose. To confirm the absence of glucose, we employed ¹H NMR spectroscopy, a sensitive and reliable method for metabolite detection. Verifying the glucose content was critical to validate the integrity of our metabolic priming approach and to ensure that any observed cellular adaptations could be confidently attributed to the intended changes in carbohydrate availability.

**Figure 3:**
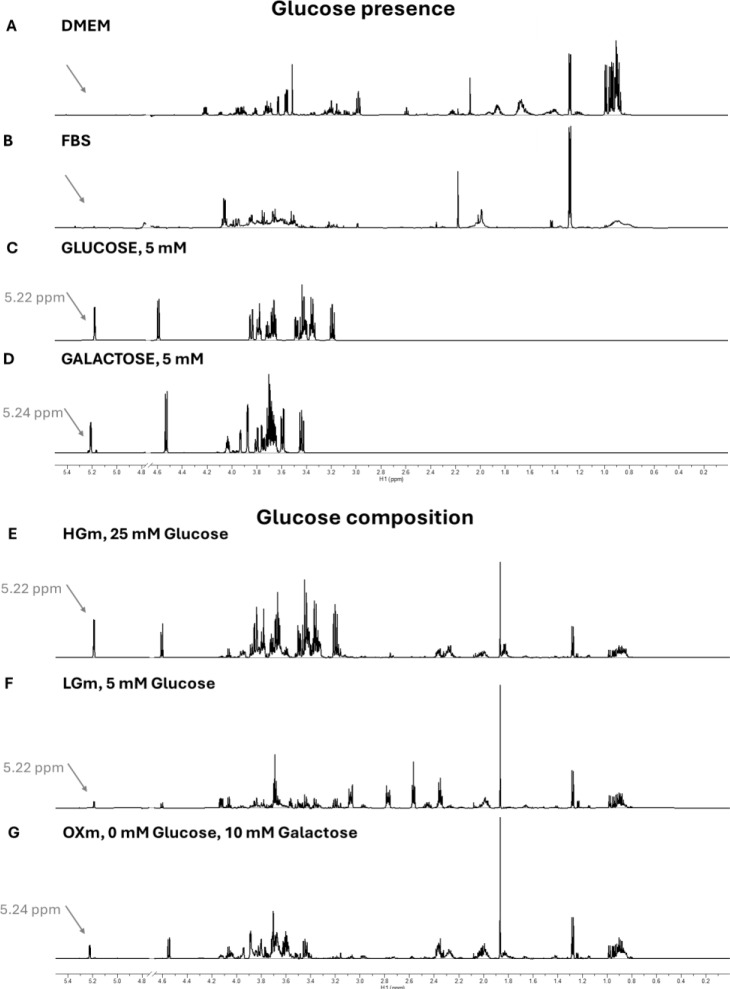
Validation of glucose absence in culture medium components and sugar composition confirmation of prepared culture media before contact with cells using ¹H NMR spectroscopy. Basal medium DMEM, fetal bovine serum (FBS) and standards of glucose and galactose at the same concentration, 5 mM were measured by NMR. This panel depicts representative 0.2-5.4 ppm region of ^1^H NMR spectra demonstrating that basal medium (A) or FBS (B) do not contain glucose or galactose demonstrated by the absence of the well resolved doublets at 5.22 ppm (J_H-H_ = 3.67 Hz) in glucose standard. 5 mM (C) or at 5.24 ppm (J_H-H _= 3.85 Hz) in the galactose standard, 5 mM (D) consistent the hydrogen 1 α signals, respectively. ^1^H NMR spectra of culture medium, HGm (E), LGm (F), and OXm (G), without contact with cells are shown. This panel illustrates the representative 0.2-5.4 ppm region of ^1^H NMR spectra revealing the presence of glucose in HGm and LGm and the presence of galactose in OXm. The well resolved doublet at 5.22 ppm (J_H-H _= 3.67 Hz) in the culture media HGm and LGm is consistent with that of the glucose hydrogen 1α and a well resolved doublet at 5.24 ppm (J_H-H _= 3.85 Hz) in the culture medium OXm is consistent with that of the galactose hydrogen 1 α.

Our NMR analyses demonstrated that basal medium and the FBS are glucose-free (Figure 3(Fig. 3), A and B, respectively). Furthermore, we confirmed that the HGm and LGm display glucose peak intensities consistent (Figure 3(Fig. 3), E and F, respectively) with standard formulations (Figure 3(Fig. 3), C). Similarly, the galactose-supplemented medium (OXm) showed peak intensity (Figure 3(Fig. 3), G) consistent with standard formulations (Figure 3(Fig. 3), D), validating its composition. These findings provided a robust foundation for the subsequent cellular studies.

**Figure 4:**
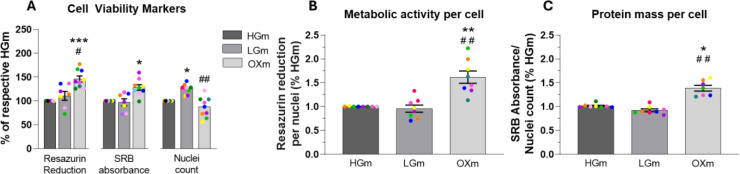
Assessment of metabolic activity and cell mass reveals enhanced metabolic profile in OXm-cultured cells. NHDF cells were plated at a density of 3750 cells/well and after 48 h cells metabolic activity and cell mass were measured through resazurin reduction assay (fluorimetry) and SRB absorbance respectively. Data represents 7-9 independent experiments and are expressed as % of HGm (A) with mean ± SEM. Values of relative fluorescence units (RFU) and optical density (from the same experiment of resazurin reduction and SRB, respectively) without normalization is represented in the supplementary information. Symbols # (against LGm) and * (against HGm). *** p<0.0005, **/## p<0.005, */# p<0.05 using Kruskal-Wallis test with Dunn's multiple comparisons test.

To further characterize the metabolic profile of fibroblasts under different culture conditions, we assessed cell mass and metabolic activity (shown in Figure 4(Fig. 4)). These parameters, when evaluated together, provide complementary insights into cell viability, proliferation, and overall metabolic status. Changes in metabolic activity relative to cell mass can reveal shifts in energy demand or mitochondrial function, thereby offering a more accurate understanding of how cells adapt to altered nutrient availability.

**Figure 5:**
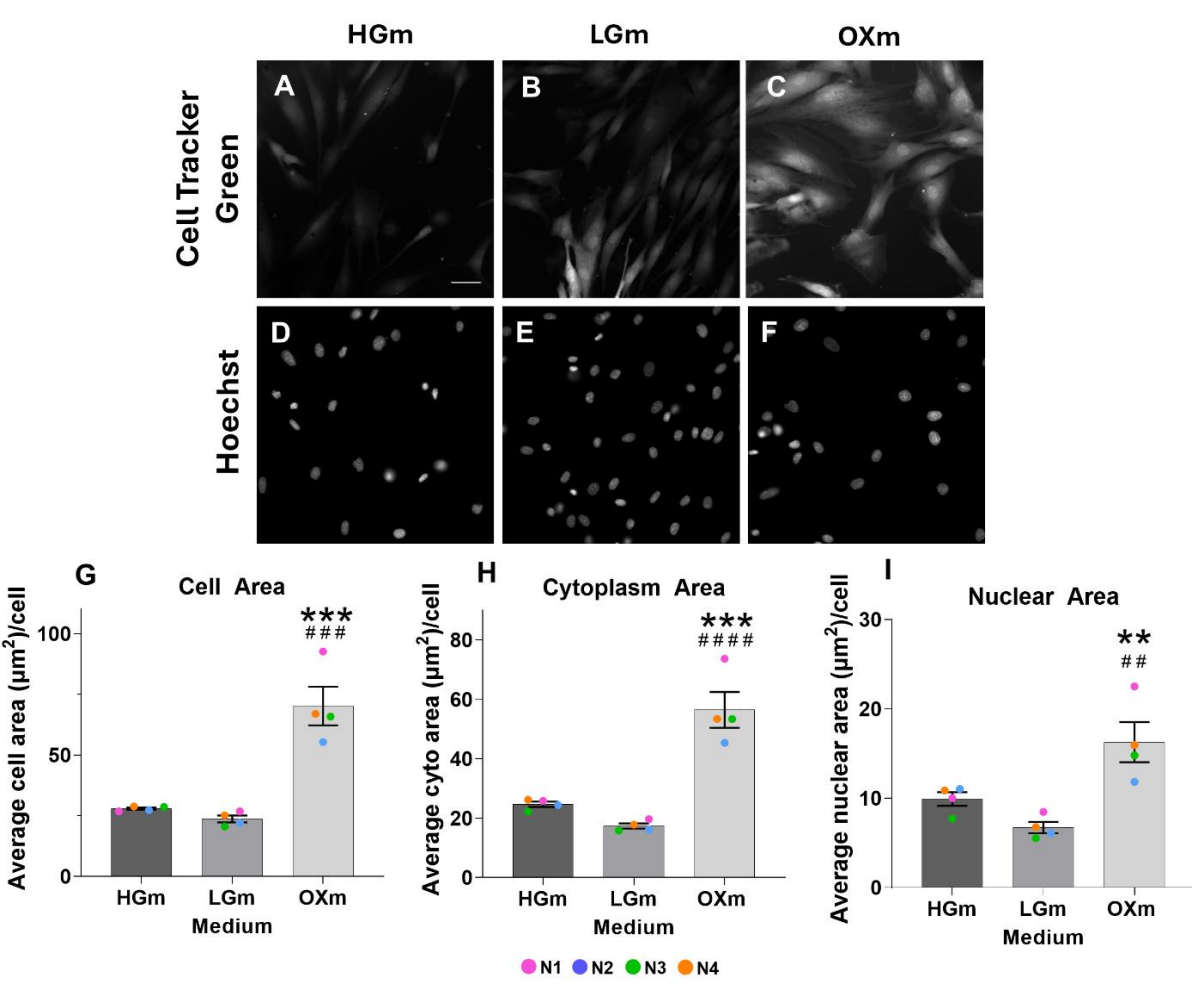
OXm-cultured cells exhibit phenotypic adaptations reflected in increased cellular areas. NHDF cells were plated at a density of 3750 cells/well and after 48 h cells were labelled with CellTracker™ Green CMFDA (CTG) dye and cells were imaged in an IN Cell Analyzer 2200. Fluorescence images were taken with the FITC-FITC2 channel (excitation 475/28 nm; emission 525/48 nm) for CTG dye (A,B,C); DAPI channel for Hoechst 33342 (D,E,F) in HGm, LGm, OXm respectively. (G, H, I), is the average of cell, cytoplasm and nuclear area (µm^2^), respectively in HGm, LGm, and OXm. Scale bar 10 µm. 1 pixel = 0.4875 µm, Data represent 4 independent experiments (each dot with a different color) and are expressed as mean ± SEM. Symbols, # against LGm and * against HGm #### p<0.0001,***/### p<0.0005, **/## p<0.005, using Kruskal-Wallis test with Dunn's multiple comparisons test.

Given the observed increase in both metabolic capacity and cell mass in cells cultured in OXm, along with the increase of the mitochondrial network in OXm, and also in the LGm conditions (Pinho et al., 2022[38]), we next investigated potential cellular phenotypic changes (Figure 5(Fig. 5)). For this purpose, we used CellTracker™ Green (CTG), a cytoplasmic dye that enables the estimation of the total cellular area by fluorescence microscopy. CTG is well-suited for this analysis due to its stable incorporation into the cytoplasm of live cells and minimal nuclear labeling, providing consistent fluorescence for cell area measurements (Breuls et al., 2003[5]).

Nuclei were counterstained with Hoechst dye to facilitate segmentation and quantification of nuclear regions. A fluorescence intensity threshold was applied to the CTG channel to generate a binary mask corresponding to labeled cells. This allowed measurement of the total area occupied by CTG-stained cells. Nuclear segmentation was performed using the Hoechst channel via the IdentifyPrimaryObjects module in CellProfiler. Hoechst staining provides strong nuclear contrast, enabling reliable detection without manual thresholding. Cytoplasmic area was determined by subtracting the nuclear area from the total cellular area.

**Figure 6:**
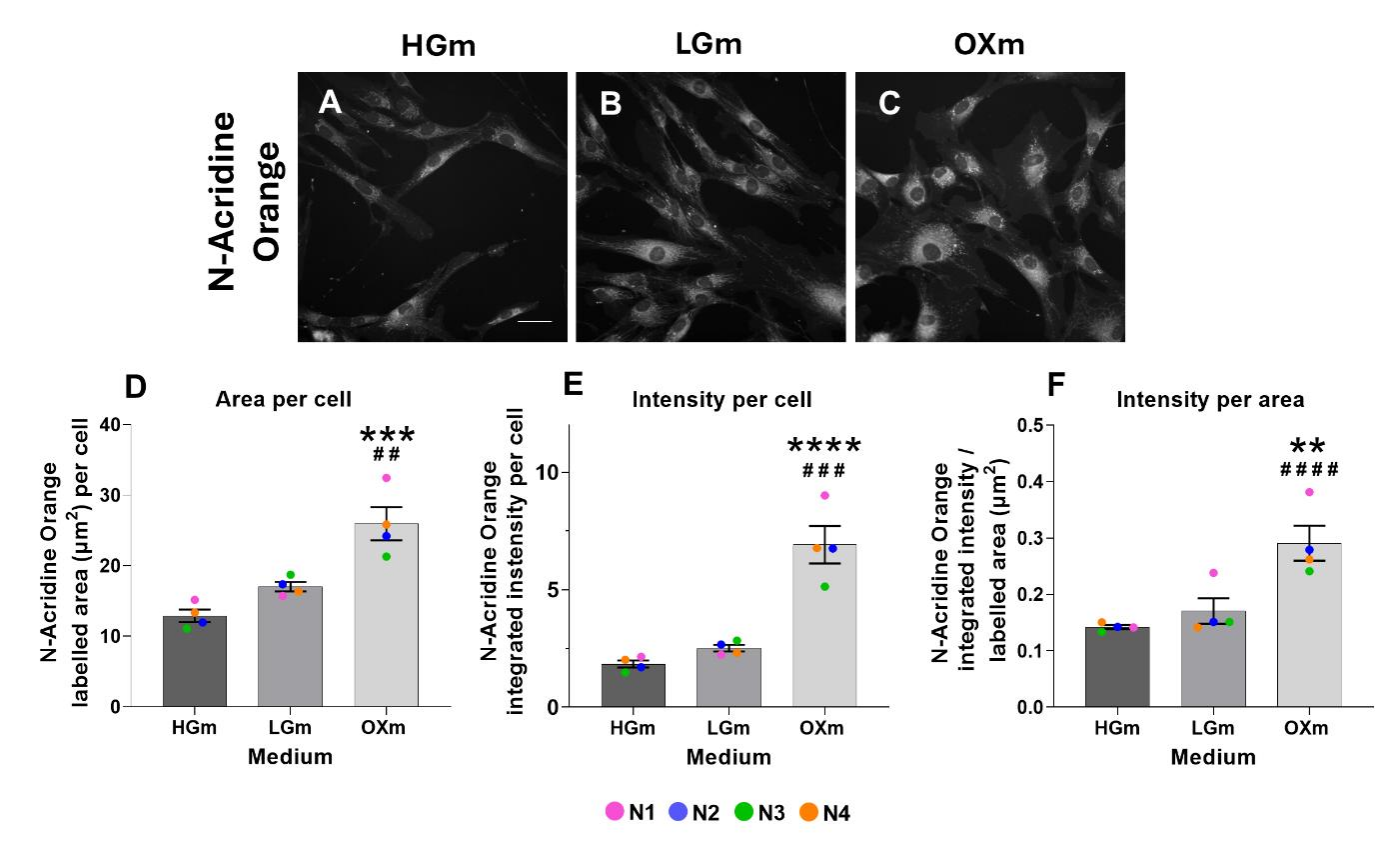
Increased mitochondrial area and cardiolipin labeling-associated fluorescence in glucose-free conditions. NHDF cells were plated at a density of 3750 cells/well and after 48 h cells were labelled with 10-N-nonyl acridine orange (NAO) for mitochondrial area and imaged in an IN Cell Analyzer 2200. Fluorescence images were taken with the FITC-FITC2 channel A, B, C in HGm, LGm, OXm respectively. D, E, F is the average of NAO-labeled area per cell, integrated intensity per cell, and integrated intensity per labeled area in HGm, LGm, and OXm. Scale bar 10 µm. 1 pixel = 0.4875 µm. Data represent 4 independent experiments (each dot with a different color) and are expressed as mean ± SEM. Symbols, # against LGm and * against HGm ####/**** p<0.0001,***/### p<0.0005, ##/** p<0.005, using Kruskal-Wallis test with Dunn's multiple comparisons test.

Given the observed increase in cellular size and metabolic activity, we next investigated mitochondrial remodeling by assessing changes in mitochondrial content and membrane composition. To this end, we used a cardiolipin-sensitive probe (nonyl acridine orange, NAO), which binds specifically to cardiolipin in the inner mitochondrial membrane and serves as a marker for mitochondrial mass and structural integrity (Figure 6(Fig. 6)). In our previous work, mitochondrial structure and function were evaluated using MitoTracker Red and TMRM, which respectively label mitochondria in a membrane potential-dependent manner. While MitoTracker Red provides high-resolution imaging of mitochondrial networks, especially when used with confocal microscopy, and TMRM allows dynamic assessment of mitochondrial membrane potential in live cells, both dyes depend on intact mitochondrial function for signal retention (Little et al., 2020[20]; Desai et al., 2024[7]). In contrast, NAO binds specifically to cardiolipin, a phospholipid localized in the inner mitochondrial membrane, making it a valuable tool for assessing mitochondrial mass and area regardless of membrane potential status (Maftah et al., 1989[26]). However, this is a controversial issue, since some evidence suggest that NAO accumulation is dependent on mitochondrial membrane potential (Keij et al., 2000[16]). Despite these potential caveats, NAO was selected for this study to enable quantification of mitochondrial area across varying metabolic or stress conditions, since we considered that could complement our previous results with TMRM (Pinho et al., 2022[38]).

We first assessed the labelled mitochondrial area per cell (A), which can provide an estimation of mitochondrial area relative to cell size. Then, total NAO fluorescence per cell was analyzed based on an estimation of cardiolipin content across the mitochondrial network (B), and finally, to distinguish between the increase in mitochondrial network and cardiolipin content, we examined mean integrated intensity per labeled cell area (C) since higher intensity in stained cells can be associated with higher cardiolipin content per cell and area (Petit et al., 1992[36]; Kaewsuya et al., 2007[15]).

**Figure 7:**
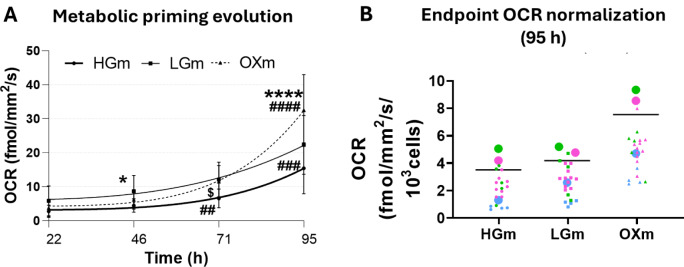
OCR nearly doubled in 24 h when cells first transitioned to 100 % OX medium. (A) OCR was measured using the Resipher device (Lucid Scientific). Cells were seeded at a density of 2000 cells per well in a Resipher plate with 75 % HGm and 25 % final medium on day 1. Over the following four days (totaling 95 h), the medium was gradually shifted: on day 2 to 50 % HGm/50 % final medium; on day 3 to 25 % HGm/75 % final medium; and on day 4 to 100 % of each final medium (HGm, LGm, or OXm). For analysis, we used the median of four OCR readings taken during the last hour before each medium change - specifically at 22, 46, 71, and 95 h after seeding. OCR was measured in real time, and the values shown in the graph correspond to the time points immediately before each medium change. (B) OCR after 95 h of culture in 100 % final medium. Data represent three independent experiments, each with 4-10 technical replicates (wells), and are presented as mean ± SEM. Statistical comparisons are indicated as # vs. LGm, * vs. HGm, $ vs. OXm. ####/**** p < 0.0001, **p<0.005, */$p<0.05. Statistical analysis was performed using the Kruskal-Wallis test followed by Dunn's multiple comparisons test. Panels A and B present data previously published by us (Pinho et al., 2025 40) which has been re-analyzed.

Cells cultured in OXm exhibited pronounced changes not only in their metabolic profile but also in their overall phenotype, including marked alterations in cellular and mitochondrial morphology. These observations raise an important question: what dynamic changes occur during the metabolic preconditioning phase, as cells transition from one nutritional environment to another? To address this, we analyzed OCR, a primary indicator of bioenergetic reprogramming. Rather than focusing solely on the endpoint of adaptation, we monitored OCR in real time throughout the metabolic transition, providing a temporal view of the cellular response to altered glucose availability (Figure 7(Fig. 7)). Following a protocol previously established and published by us (Pinho et al., 2025[39]), we measured the real-time OCR using the Resipher device (Figure 7(Fig. 7) A, B). Interestingly, within the first 24 h of transitioning to 100 % OXm, OCR nearly doubled (Pinho et al., 2025[39]). 

**Figure 8:**
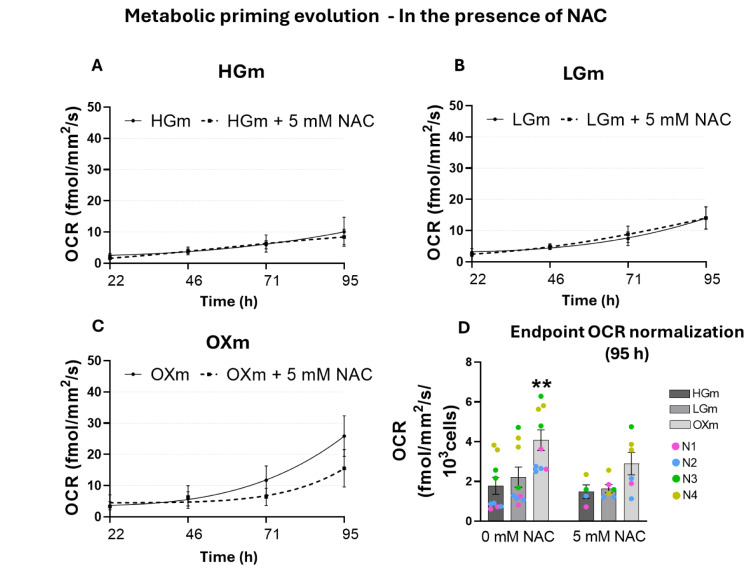
Real-time bioenergetic profiling reveals ROS-mediated metabolic adaptation during medium transition. We used NAC as a strategy to underscore if reactive species are involved in this bioenergetic adaptation (Figure 7). Cells were seeded as described above, with the addition of 5 mM NAC in some conditions on day 1. Medium was changed daily - always in the presence or absence of NAC - to 25 %, 50 %, 75 % and finally 100 % of the respective final medium (HGm, LGm, or OXm) by day 4. Median OCR values were again calculated from the last hour before each medium change, at 22, 46, 71, and 95 h. Real-time OCR measurements were taken, and final values were extracted immediately before medium changes for HGm (A), LGm (B), and OXm (C). (D) OCR after 95 hours of culture in 100 % final medium, normalized per 10³ cells. Data represent four independent experiments, each with 4-10 technical replicates (wells), and are presented as mean ± SEM. Statistical comparisons are indicated as **p<0.005 against HGm. Statistical analysis was performed using the Kruskal-Wallis test followed by Dunn's multiple comparisons test.

Given that our previous work (Pinho et al., 2022[38]) showed increased reactive species (RS) levels in this medium, we questioned whether RS might be driving this metabolic rewiring. To explore this possibility, we monitored OCR during the metabolic priming process in the presence of the antioxidant NAC, which was used as a strategy to study the effect of RS in the cellular adaptation (Figure 8(Fig. 8) A-D). Importantly, while previous studies - including our own - focused on endpoints after adaptation (e.g. bioenergetic analysis in NHDF cells evaluated by the Seahorse^TM^ Mito stress test (Pinho et al., 2022[38])), the dynamic understanding of how cells adjust throughout the adaptation process had not been addressed. This real-time analysis represents a significant advantage, allowing us to observe cellular responses as they unfold. We hypothesized that the increased RS production, likely resulting from elevated mitochondrial activity, could be an active contributor to the adaptation process itself.

See also the supplementary information.

## Discussion

In this study, we characterized the metabolic shifts that occur as cells transition from high to low or absent glucose concentrations in the culture medium. Beyond mapping these changes in metabolic activity, we evaluated their impact on cellular morphology and investigated the key factors driving this metabolic rewiring within the context of our metabolic priming model. This work showed that culturing human skin fibroblasts in different metabolic conditions impacted not only mitochondrial metabolism and activity (Pinho et al., 2022[38]) but also cellular morphology, structural organization, and functional state (Figure 4(Fig. 4), 5(Fig. 5) and 6(Fig. 6)). In our study, we evaluated cell viability/mass and metabolic function across media conditions using three key indicators: Resazurin reduction, Sulforhodamine B (SRB) absorbance, and nuclei count. Notably, cells cultured in OX medium (devoid of glucose) exhibited significantly higher Resazurin reduction and SRB absorbance compared to other groups, HGm and LGm, despite a lower total nuclei count. This paradox suggests that while fewer cells are present, each cell maintains (or even increases) its metabolic activity and protein content. Resazurin is a redox-sensitive dye reduced to fluorescent resorufin by intracellular dehydrogenases. These enzymes are primarily located in the cytoplasm but also exist in mitochondria (Petit et al., 1992[36]; O'Brien et al., 2000[32]; Lavogina et al., 2022[17]). The reduction of Resazurin to resorufin is driven by the availability of reducing equivalents, particularly NADH and NADPH, which function as both products and regulators of cellular metabolism (Lavogina et al., 2022[17]). The elevated Resazurin/nuclei ratio in OX medium thus reflects enhanced metabolic activity per cell, possibly indicating increased mitochondrial respiration or compensatory upregulation of oxidative pathways in the absence of glycolytic input, findings that align with the previously reported increase in OCR (Pinho et al., 2022[38]) and in the present work (Figure 6(Fig. 6), 7(Fig. 7) and 8(Fig. 8)), in the OXm cells. In contrast, SRB binds stoichiometrically to basic amino acid residues of cellular proteins under mildly acidic conditions. It serves as a proxy for total protein mass, correlating with cell size and biosynthetic activity (Vichai and Kirtikara, 2006[48]; Silva et al., 2016[45]). The increased SRB/nuclei ratio observed in OX medium suggests that, although cell proliferation is reduced (as indicated by a lower number of nuclei), individual cells exhibit higher protein content - consistent with a more metabolically active or differentiated phenotype. This observation aligns with previous reports showing that nutrient deprivation, particularly glucose restriction, promotes a shift toward mitochondrial oxidative phosphorylation and triggers cellular remodeling. Such changes may underlie further phenotypic adaptations. Collectively, our data indicates that glucose removal in OX medium induces a state of reduced proliferation accompanied by enhanced metabolic and biosynthetic activity at the single-cell level. This condition likely favors oxidative metabolism, as evidenced by increased mitochondrial function and protein content normalized to cell number.

This rationale supports our subsequent analyses using CTG and NAO to investigate the increase in cell mass, changes in cell morphology and mitochondrial mass under these metabolic conditions.

In the absence of glucose, the increase in mitochondrial network (higher mitochondrial area per cell (Pinho et al., 2022[38]) was accompanied by an increase in metabolic activity (Figure 4(Fig. 4)) and overall cellular and subcellular size, such as cytoplasm and nuclei (Figure 5(Fig. 5) and 6(Fig. 6)). In fact, the higher summed branches length in OXm cells (higher mitochondrial connectivity) happens when cells are more dependent on oxidative phosphorylation for energy production, when nutrients (e.g. glucose) are limited (Pinho et al., 2022[38]). This higher interconnected mitochondrial network functions as an internal transport system, ensuring efficient energy delivery and a metabolic support across greater cellular distances, when cells are bigger (Miettinen and Bjorklund, 2017[29]), as it is the case of OXm cells. These results reinforce the interplay between mitochondrial and cell area with energy metabolism (Pinho et al., 2022[38]). Cell size is a critical determinant of cellular function, influencing key processes such as biosynthesis, metabolism, and nutrient uptake. Its regulation is often tightly coordinated with cell growth and progression through the cell cycle (Fung and Bergmann, 2023[11]). Cell size is tightly regulated across diverse organisms, with size checkpoints proposed to control key transitions in the cell cycle, particularly the G1/S and G2/M phases (Echave et al., 2007[10]; Liu et al., 2024[23]). In line with these observations, our previous study revealed an altered G2/M checkpoint profile in cells cultured in OX medium, marked by increased heterogeneity and the emergence of two distinct subpopulations - one exhibiting elevated, and the other reduced, G2/M phase frequency (Pinho et al., 2025[40]). Notably, the subpopulation with reduced G2/M frequency exhibited higher mitochondrial membrane potential, suggesting a possible link between mitochondrial activity and checkpoint regulation. These findings highlight the potential contribution of metabolic state and mitochondrial function to cell cycle dynamics and cell size homeostasis under nutrient-restricted conditions (Miettinen and Bjorklund, 2017[29]). These findings also raise the possibility that OXm-cultured cells may be undergoing senescence. Cellular senescence is often accompanied by increased cell size, altered mitochondrial dynamics, and cell cycle arrest, particularly in G1 or G2/M phases (Neurohr et al., 2019[31]). Mitochondrial activity and oxidative stress are known to contribute to senescence induction (Ghosh-Choudhary et al., 2021[12]). Therefore, the metabolic and cell cycle alterations observed in OX medium could reflect, at least in part, a shift toward a senescent phenotype in those cells. However, this hypothesis warrants further investigation using established senescence markers such as SA-β-gal activity, p21/p16 expression, or DNA damage *foci *(Rodier and Campisi, 2011[41]). 

Interestingly, the increase in mitochondrial area and intensity assessed through NAO-stained cells (Figure 6(Fig. 6)) may indicate a rearrangement of phospholipid membranes in cells cultured in OXm, since NAO binds specifically to cardiolipin (Rodriguez et al., 2008[42]), an essential constituent of mitochondrial membranes, playing a co-factor role in several mitochondrial functions, including respiration (Dudek, 2017[9]; Paradies et al., 2019[34]). This supports our previous findings of increased TMRM-labeled area, indicative of a more extensive and polarized mitochondrial network (Pinho et al., 2022[38]). Notably, NAO and TMRM serve as complementary probes - while NAO reflects the lipid mass of mitochondria by targeting cardiolipin, TMRM assesses mitochondrial membrane potential, offering insights into functional activity. NAO has been used to evaluate mitochondrial content showing a good correlation between NAO fluorescence and mitochondrial area in mouse fibroblasts and hybrid cell lines (Lizard et al., 1990[24]) and in hepatocytes from adult male Wistar rats (Petit et al., 1992[36]). Also to monitor mitochondrial membrane biosynthesis of L1210 cells, NAO fluorescence was used to assess changes in mitochondrial mass, providing insights into mitochondrial biogenesis in relation to the cell cycle (Leprat et al., 1990[18]).

To gain deeper insight into the mechanisms underlying cellular and bioenergetic adaptation - and in light of the elevated oxidative stress previously observed in OXm-cultured cells - we employed NAC to test the hypothesis that RS are key drivers of mitochondrial and cellular remodeling in response to distinct metabolic environments. NAC is widely used in both *in vitro* and *in vivo* models due to its well-characterized antioxidant properties and its ability to mitigate oxidative stress (Pedre et al., 2021[35]). There are three potential hypothesis to explain the biological effects of NAC, namely: disulfide reduction (ability to reduce disulfide bonds both outside and inside cells, which may play a crucial role in protein folding, structure, and function); oxidant scavenging (e.g. H_2_O_2_, HOCl (hypochlorous acid), ^•^OH); and glutathione replenishment (restoring or increasing levels of glutathione in cells is essential for maintaining effective antioxidant defense and cellular health) (Pedre et al., 2021[35]). 

Using the Resipher device (Figure 7(Fig. 7)), we monitored OCR in real time and observed a striking ~2-fold increase when cells were transitioned to 100 % OXm. This finding suggests that only in the complete absence of glucose do cells become fully reliant on mitochondrial oxidative phosphorylation for ATP production, resulting in a significant elevation in mitochondrial respiration. Notably, when the medium transition was carried out in the presence of NAC (Figure 8(Fig. 8)), the OCR increase observed in OXm was markedly attenuated. This supports the hypothesis that the shift toward mitochondrial dependence in OXm - absent in both LGm and HGm conditions - is mediated, at least in part, by reactive redox species, as evidenced by the suppressive effect of NAC (Figure 8(Fig. 8)A-C). This effect may be linked to alterations in the cellular redox state, as NAC serves as a precursor for reduced glutathione and may influence Nrf2-mediated expression of antioxidant response genes (Mokhtari et al., 2017[30]; Aldini et al. 2018[1]; Pedre et al., 2021[35]; Sahasrabudhe et al., 2023[44]). Also, NAC can indirectly interact with pentose phosphate pathway (PPP) pathway, with NADH and GPx equilibrium (Xiao et al., 2018[50]). In human gingival fibroblasts (HGFs) subjected to oxidative stress induced by H₂O₂, treatment with NAC reduced mitochondrial oxygen consumption and increased non-mitochondrial respiration, suggesting a cellular shift toward alternative oxygen-utilizing pathways. Interestingly, in the same study, resveratrol demonstrated a greater antioxidant capacity than either NAC or quercetin (Orihuela-Campos et al., 2015[33]).

The reduction or elimination of an observable effect - such as decreased OCR following NAC treatment (Figure 7(Fig. 7)) - is commonly interpreted as evidence that RS mediate the phenomenon under investigation, including cellular adaptation to different metabolic environments, as discussed in this work (Pedre et al., 2021[35]). 

## Conclusion

These findings highlight the intricate interplay between metabolic context, cellular morphology, and oxidative stress in NHDF cells. The shift from glycolysis to OXPHOS, triggered by reduced glucose availability, induced marked changes in cellular metabolism, growth dynamics, and morphology. Notably, the increase in cell size observed under OXm conditions may reflect features characteristic of a senescence-like phenotype. This supports the potential use of metabolically primed NHDFs as a valuable *in vitro* model for studying mechanisms underlying cellular senescence and aging-related pathologies, potentially complicated by the presence of co-morbidities. Our data also emphasize that increased cell size can significantly influence the outcomes of various experimental assays, underscoring the importance of appropriate normalization strategies. In proliferative and mononucleated cell cultures, normalizing experimental results to the number of nuclei - rather than total cell mass - provides a more accurate representation of cellular responses, accounting for differences in both cell size and proliferation rates. Finally, the suppression of mitochondrial activation by the antioxidant NAC underscores the role of redox species as key signaling mediators in metabolic adaptation. These insights advance our understanding of cellular responses to metabolic stress and may inform the development of targeted strategies to modulate cellular metabolism in aging, disease, and therapeutic interventions.

## Declaration

### Acknowledgements

We thank to João Patrício for the help with the NMR measurements.

The authors' laboratories were funded by the European Regional Development Fund (ERDF), through the Centro 2020 Regional Operational Programme under projects CENTRO-01-0246-FEDER-000010 (Multidisciplinary Institute of Ageing (MIA-Portugal, in Coimbra) through the COMPETE 2020 - Operational Programme for Competitiveness and Internationalisation and Portuguese national funds via FCT - Fundação para a Ciência e a Tecnologia, under projects UIDB/04539/2020, UIDP/04539/2020 and LA/P/0058/2020. SAP and TCO were funded by SFRH/PD/BD/143055/2018 and DL57/2016/CP1448/CT0016. This work was also supported by European Union's Horizon Europe programme project PAS GRAS under Grant Agreement 101080329. Views and opinions expressed are however those of the author(s) only and do not necessarily reflect those of the European Union or the European Commission. Neither the European Union nor the granting authority can be held responsible for them. 

### Conflict of interest

The authors declare no conflict of interest.

### Author contributions

S.A.P. performed the experiments, analyzed the data, and wrote the paper. C.B. and J.G.J. measured the samples in the NMR equipment and helped with all the procedure. C.D. performed measurement of mitochondrial area. T.C.O. analyzed the CellProfiler data; T.C.O. and P.J.O. designed research, corrected the manuscript and acquired funding. All authors contributed to the final version of the manuscript.

### Disclaimers

This work benefited from the use of artificial intelligence (AI)-powered language models to improve readability and clarity. All AI-assisted text was reviewed and edited by the authors, who assume full responsibility for the content of the manuscript.

### Data sharing statement

The dataset generated and analyzed during the current study are available on doi: 10.6084/m9.figshare.29618663. 

### ORCIDs

Sónia A. Pinho, ORCID: 0000-0002-7426-5197; Cristina Barosa, ORCID: 0000-0001-7819-2434; Cláudia Deus, ORCID: 0000-0002-8344-9182; John Griffith Jones, ORCID: 0002-3745-3885; Paulo J. Oliveira, ORCID: 0000-0002-5201-9948; Teresa Cunha-Oliveira, ORCID: 0000-0002-7382-0339.

## Supplementary Material

Supplementary information

## Figures and Tables

**Figure 1:**
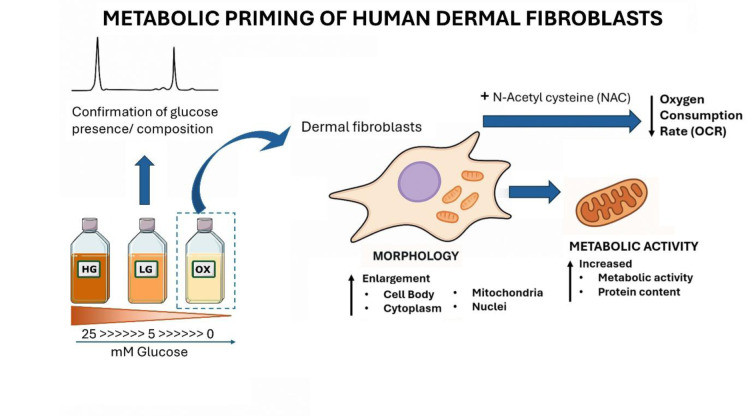
Graphical abstract
