# Supplementary material for: Metabolic priming alters the morphology and metabolism of human dermal fibroblasts
Source: EXCLI J. 2025 Oct 22;24:1419–37. doi: 10.17179/excli2025-8609 (PMC12598107; doi:10.17179/excli2025-8609)
Supplement: Supplementary information [file EXCLI-24-1419-s-001.pdf]

EXCLI Journal 2025;24:1419-1437 – ISSN 1611-2156
Received: May 31, 2025, accepted: July 29, 2025, published: October 22, 2025

Supplementary information to:

Original article:

METABOLIC PRIMING ALTERS THE MORPHOLOGY AND METABOLISM OF HUMAN DERMAL FIBROBLASTS

Sónia A. Pinho[1,2,3], Cristina Barosa[1,2], Cláudia M. Deus[2,4], John G. Jones[1,2], Paulo J. Oliveira[1,2], Teresa Cunha-Oliveira[1,2]

[1] CNC – UC, Center for Neuroscience and Cell Biology, University of Coimbra, Portugal
[2] CIBB - Center for Innovative Biomedicine and Biotechnology, University of Coimbra, Portugal
[3] PhD Program in Experimental Biology and Biomedicine (PDBEB), Institute for Interdisciplinary Research (IIIUC), University of Coimbra, Portugal
[4] MIA-Portugal, Multidisciplinary Institute of Ageing, University of Coimbra, Portugal

* **Corresponding author:** Paulo Oliveira, Ph.D., CNC – UC, Center for Neuroscience and Cell Biology, University of Coimbra, Portugal; CIBB - Center for Innovative Biomedicine and Biotechnology, University of Coimbra, UC Biotech Building, Lot 8A, Biocant Park, 3060-197 Cantanhede, Portugal. Phone: +351-231-249-195, fax: +351-231-249-179; E-mail: pauloliv@cnc.uc.pt

https://dx.doi.org/10.17179/excli2025-8609

This is an Open Access article distributed under the terms of the Creative Commons Attribution License (https://creativecommons.org/licenses/by/4.0/).

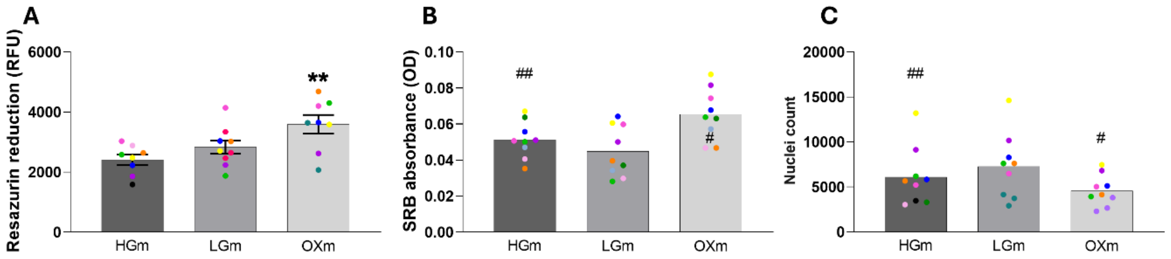

***Supplementary Figure 1*: Assessment of metabolic activity and cell mass reveals enhanced metabolic profile in OXm-cultured cells and higher protein content per cell in OXm cells.** NHDF cells were plated at a density of 3750 cells/well and after 48 h cells metabolic activity (**A**), cell mass (**B**) and nuclei count (**C**) were measured through resazurin reduction assay (fluorimetry), SRB absorbance and nuclei stained with Hoechst 33342, respectively. Data represents 7-9 independent experiments and are expressed as values of relative fluorescence units (RFU), optical density and the counted nucleus (from the same experiment of resazurin reduction and SRB, respectively) without normalization to show the variability between experiments. Each experiment is represented with a different color (the same for A, B and C). Symbols, # (against LGm) and * (against HGm): ** /## p<0.005, # p<0.05 using Šídák's multiple comparisons test.
